# Retracted: Hydrostatin-SN10 Ameliorates Pancreatitis-Induced Lung Injury by Affecting IL-6-Induced JAK2/STAT3-Associated Inflammation and Oxidative Stress

**DOI:** 10.1155/2021/8541783

**Published:** 2021-01-19

**Authors:** Oxidative Medicine and Cellular Longevity

*Oxidative Medicine and Cellular Longevity* has retracted the article titled “Hydrostatin-SN10 Ameliorates Pancreatitis-Induced Lung Injury by Affecting IL-6-Induced JAK2/STAT3-Associated Inflammation and Oxidative Stress” [1] at the request of the authors due to not acknowledging prior work and errors in the analysis.

Hydrostatin-SN10, derived from Hydrostatin-SN1, was previously described in two Master's theses that were not cited [2, 3]. Patents held by Yiming Lu and colleagues [4, 5] were not acknowledged and the peptide supplier had a confidentiality agreement with Dr. Lu.

The KD value for SN10 given in the introduction has the wrong unit (*μ* versus *μ*M) and cannot be relied upon because it was not validated by the use of surface plasmon resonance (SPR) analysis [6] or isothermal titration calorimetry (ITC) [7].

Two control groups should have been included, as noted in [8]: a positive control in the group treated by the SN10 mutant and a negative control in the group treated by the unrelated octapeptide, otherwise the results cannot be stated to have been specifically caused by SN10 treatment. Similar results may be induced by other decapeptides (for example, see [9–11]).

The authors apologized and the editorial board approved the retraction.
